# Physical Activity and Screen Time in Metabolically Healthy Obese Phenotypes in Adolescents and Adults

**DOI:** 10.1155/2013/984613

**Published:** 2013-09-11

**Authors:** Sarah M. Camhi, Molly E. Waring, Susan B. Sisson, Laura L. Hayman, Aviva Must

**Affiliations:** ^1^Department of Exercise and Health Sciences, College of Nursing and Health Sciences, University of Massachusetts, Boston, 100 Morrissey Boulevard, Boston, MA 02125, USA; ^2^Department of Quantitative Health Sciences, University of Massachusetts Medical School, Worcester, MA, USA; ^3^Department of Nutritional Sciences, College of Allied Health, University of Oklahoma, Oklahoma City, OK, USA; ^4^Department of Nursing, College of Nursing and Health Sciences, University of Massachusetts, Boston, MA, USA; ^5^Department of Public Health and Professional Degree Programs, School of Medicine, Tufts University, Boston, MA, USA

## Abstract

*Introduction*. The purpose of this study was to examine levels of physical activity (PA) and screen time (ST) in metabolically healthy obese (MHO) and metabolically unhealthy obese (MUO) adolescents and adults. *Methods*. NHANES data from obese adolescents (12–18 years, BMI *z*-score ≥ 95th percentile) and adults (19–85 years, BMI ≥ 30 kg/m^2^) were pooled from 2003–2005 cycles. Metabolic phenotypes were categorized as MHO (0 or 1 cardiometabolic risk factor; triglycerides, HDL-C, blood pressure, or glucose) or MUO (≥2 cardiometabolic risk factors). Logistic regression models estimated associations between phenotype and PA/ST adjusted for age, gender, BMI, race/ethnicity, menopausal status, and NHANES cycle. *Results*. Among adolescents, PA was not associated with MHO. In contrast, MHO adults 19–44 years were 85% more likely to engage in active transportation and 2.7 times more likely to be involved in light intensity usual daily activity versus sitting. For each minute per day, adults 45–85 years were 36% more likely to have the MHO phenotype with higher levels of moderate PA. ST was not associated with metabolic phenotypes in adolescents or adults. *Conclusion*. The current study provides evidence that PA, but not ST, differs between MHO and MUO in adults, but not in adolescents. Future studies are needed to confirm results.

## 1. Introduction

Obesity is recognized as a heterogeneous condition whereby some obese adolescents and adults have an unfavorable cardiometabolic phenotype (metabolically “unhealthy” obese, MUO), while others have a lower risk phenotype (metabolically “healthy” obese, MHO) [1–5]. MHO are characterized by individuals who exhibit favorable lipid, insulin, and blood pressure levels [1, 6, 7] despite the presence of obesity. MHO adults have lower incidence of cardiovascular disease and type II diabetes compared to MUO adults [8], thus, MHO may represent a reduced level of chronic disease risk within obesity. Approximately 65% of obese U.S. adolescents have the MHO phenotype [5], while 32% of obese adults have the MHO phenotype [9]. Thus, a shift of cardiometabolic risk from MHO towards the “unhealthy” phenotype appears to occur during the transition from adolescence to adulthood. 

Population studies suggest that higher levels of physical activity and less sedentary behavior reduce the risk of obesity and lower cardiometabolic risk [10–12]. These lifestyle behaviors could differentiate MHO and MUO phenotypes and may provide targets for intervention strategies to modify and lower cardiometabolic risk among obese individuals. Although physical activity was not associated with the MHO phenotype among postmenopausal, sedentary, obese women [1, 13], population-based studies of obese men and women have documented associations of higher levels of physical activity with the MHO phenotype in both the U.S. [9] and Switzerland [14]. However, previous studies have not investigated specific qualities of physical activity which have known positive effects on cardiometabolic risk and obesity such as intensity (e.g., moderate versus vigorous) [15, 16], type (e.g., strength training) [17, 18], or domain (e.g., active transportation, occupational activity) [19–21]. In addition, these studies have been exclusively in adult populations; physical activity levels have not been examined in MHO and MUO adolescents.

Despite our current knowledge with respect to physical activity levels in MHO, few studies have characterized sedentary behaviors among these phenotypes in either adults or adolescents. Sedentary behavior can be operationally estimated as time spent sitting, using the computer or television (TV) viewing. Previous studies have shown that higher levels of sitting [22] and TV viewing [23] are related to cardiovascular risk, disease, and mortality. In addition, higher levels of screen time (TV and computer) are associated with cardiometabolic risk and obesity [23–26] in both adolescents and adults. Since screen time levels are high amongst U.S. adolescents [27] and adults [28, 29], this may be an important variable to consider for modifying risk within obesity phenotypes. 

Therefore, the purpose of this study was to examine levels of physical activity and screen time in MHO and MUO adolescents and adults.

## 2. Methods

Data from the National Health and Nutrition Examination Survey (NHANES) 2003-2004 and 2005-2006 cycles were pooled to maximize adolescent and adult sample sizes. The NHANES is a continuous 2-year health surveillance of the U.S. population where randomly selected individuals participate in a home interview, physical examination, and a laboratory exam. Detailed study procedures are available from the online manual [30]. All participants provided written informed consent, and a parent/guardian also provided informed consent for any participant less than 18 years. Procedures for data collection were approved by the National Center for Health Statistics' Institutional Review Board, and secondary data analyses were approved by the University of Massachusetts Boston Institutional Review Board. 

The home interview gathered information on self-reported sociodemographic information. Adults and adolescents who were 16 years of age or older were interviewed directly. Proxy respondents such as parents provided information for participants aged 12–15 years and for participants unable to respond to questions. 

The physical examination included the measurement of height, weight, blood pressure, and cardiovascular and other laboratory measures. Blood pressure was measured after a 5-minute rest. A maximum of four readings were obtained; consecutive readings, excluding the first measure, were averaged for this analysis. Blood was drawn after a 10-hour fast, in the left arm, at the antecubital vein. All laboratory procedures for cardiovascular risk factors were standardized.

 Body mass index (BMI, kg/m^2^) was calculated from measured height and weight. For adolescents (12–18 years), obesity was operationalized by age- and sex-specific CDC BMI percentile ≥95th percentile [31]. For adults, obesity was operationalized by a BMI ≥30 kg/m^2^. 

For adolescents, MUO was defined as 2 or more cardiometabolic risk factors: triglycerides ≥110 mg/dL or on cholesterol medication, high density lipoprotein cholesterol (HDL-C) <40 mg/dL or on cholesterol medication, blood pressure ≥90th percentile for age, gender, and height or on blood pressure medication, and fasting glucose ≥100 mg/dL or on glucose/insulin medication [32]. For adults, MUO was defined as 2 or more cardiometabolic risk factors: triglycerides ≥150 mg/dL or on cholesterol medication, HDL-C <40 mg/dL for men, <50 mg/dL for women or on cholesterol medication, blood pressure ≥130/85 mmHg or on blood pressure medication, and fasting glucose ≥100 mg/dL or glucose/insulin medication [33]. For both adolescents and adults, MHO was defined as having 0 or 1 abnormal cardiometabolic risk factors.

### 2.1. Physical Activity

Participants ≥16 years of age reported their physical activity behaviors during the household interview; adolescents 12–15 years of age answered these questions during the physical examination: (1) “over the past 30 days, did you do any vigorous activities for at least 10 minutes that cause heavy sweating, or large increases in breathing or heart rate?” and (2) “over the past 30 days, did you do moderate activities for at least 10 minutes that cause only light sweating or a slight to moderate increase in breathing or heart rate?”. If the respondent answered “yes” for either question, additional questions were asked to characterize the type of activity, intensity, number of times performed over past 30 days, number of minutes the activity was performed. These variables then were summed to yield total daily minutes of moderate physical activity (MPA), vigorous physical activity (VPA), and combined for moderate-to-vigorous physical activity (MVPA) in minutes per day (mins/day). Data were also expressed as total physical activity (total PA, MET ∗ mins/week) by multiplying the frequency, intensity (in METS), and duration of any reported MVPA activity. 

Participants met the recommended levels for physical activity if MVPA ≥ 60 minutes of MVPA per day for adolescents and ≥30 minutes of MVPA per day for adults, in order to represent a goal equivalent to the National Guidelines for Physical Activity [34]. 

Additional questions asked participants to report how often (frequency or number of times) over the past 30 days they engaged in physical activities specifically designed to strengthen muscles (i.e., lifting weights, push-ups, or sit-ups); which was then averaged into the number of days per week. 

Adolescents and adults were asked if they had walked or bicycled for transportation over 30 days (yes or no, reported as % currently engaged with active transport). They were also asked about the frequency and duration of engagement in active transportation over the past 30 days, and these values were made equivalent to time spent in minutes in active transportation per week in mins/week.

Usual daily activity, which can include typical activity done at work, school, or home, was assessed in participants ≥16 years of age and divided into 4 possible groups: mostly sitting, walking, light activity, or heavy activity. This variable was only included for adults 19–85 years of age because adolescents aged 12–15 years were not asked to estimate usual daily activity.

### 2.2. Screen Time

Adolescents and adults reported TV/video and computer use via two questions: (1) “over the past 30 days, on average, how many hours per day did you sit and watch TV or videos?” and (2) “over the past 30 days, on average about how many hours per day did you use a computer or play computer games?” Responses for computer and TV/videos were independently categorized as ≤1 hour per day, 2-3 hours per day, and ≥4 hours per day. 

Total screen time was estimated by combination of responses for both TV/video and computer use. Respondent's screen time was characterized relative to American Academy of Pediatrics (AAP) guidelines which recommend that children and adolescents have no more than 2 hours of screen time per day [35]. Although no recommendations for adults have been made for screen time, the same thresholds of ≤2 hours or less versus >2 hours of screen time were used to classify those who meet or exceed the youth recommendations, respectively.

 NHANES 2003-2004 and 2005-2006 included 335 adolescents and 635 adults 19–44 years of age and 779 adults 45–85 years of age who were obese and had a physical exam with assessment of cardiometabolic variables (triglycerides, HDL-C, systolic and diastolic blood pressure, and glucose). Those who had fasted for fewer than 10 hours for the blood draw (adolescents *n* = 50; adults 19–44 years *n* = 96, adults 45–85 years *n* = 100), were pregnant (adolescents *n* = 8; adults 19–44 years *n* = 92, adults 45–85 years *n* = 0), were diabetic (adolescents *n* = 3; adults 19–44 years *n* = 24, adults 45–85 years *n* = 154), or were missing physical activity or screen time data (adolescents *n* = 40, adults 19–44 years *n* = 143, adults 45–85 years *n* = 248) were excluded. Also excluded were subjects whose MPA or VPA values were outliers, defined as more than 3 standard deviations from the unweighted mean (adolescents *n* = 9; adults 19–44 years *n* = 10, adults 45–85 years *n* = 6). These exclusions resulted in an analytic sample of 225 adolescents and 270 adults 19–44 years, and 271 adults 45–85 years.

### 2.3. Statistical Analysis

All analyses were stratified by three age groups (adolescents, 12–18 years, and adults 19–44 years, and adults 45–85 years) in order to avoid any potential bias of age on physical activity or screen time levels. Adults were divided based on the distribution of ages in the sample at the median value. Non-normally distributed variables were log-transformed prior to statistical analyses (i.e., triglycerides, MPA, VPA, MVPA, PA, strength, and active transport). Demographic and cardiometabolic characteristics, physical activity and screen time characteristics were compared between MHO and MUO using *t*-tests for continuous variables and chi-square tests for categorical variables. 

Logistic regression models were used to estimate the association of physical activity and sedentary behavior with metabolic phenotype (MHO versus MUO), adjusting for age, gender, BMI (*z*-score in adolescents; kg/m^2^ in adults), race/ethnicity (non-Hispanic white, non-Hispanic black, Mexican American, and others: mixed and Hispanic), menopausal status, and NHANES cycle. MHO was the modeled behavior, and the lifestyle behavior (physical activity or screen time) that served as the reference group was the one that would be more desirable (i.e., highest level of physical activity or lowest level of screen time).

Analyses were performed using SAS (version 9.3, SAS Institute, Cary, NC, USA) with procedures specific to complex sampling designs, thus allowing estimates representative of obese adolescents and adults in the U.S. We included sample weights (from physical exam fasting data) and weighted the data to reflect NHANES multiple data cycles, as recommended by the National Center for Health Statistics [36]. Descriptive data are presented as means ± standard error.

## 3. Results

The prevalence of MHO among obese individuals was 68% in adolescence, 54% in adults 19–44 years of age, and 24% in adults 45–85 years of age. Those who were classified as MHO were less likely to be male in adolescents and adults 45–85 years. Adults 19–44 years with MHO were younger and had a lower BMI on average than those with MUO; however, age and BMI were not significant in adolescents or adults 45–85 years of age. No significant differences were identified between metabolic phenotypes with respect to ethnicity in any age group (Table 1). By definition, those with MHO phenotype had lower triglycerides, systolic blood pressure, fasting glucose, and higher HDL-C in all age groups (Table 1). 

Among obese adolescents, neither physical activity nor screen time variables were associated with metabolic phenotype (Table 2). Compared to MUO, adults 19–44 years of age with the MHO phenotype engaged in significantly more VPA mins/day (MHO versus MUO: 15.1 ± 1.9 versus 8.7 ± 1.6, *P* = 0.01), and a higher proportion participated in active transportation (37% versus 23%, *P* = 0.01; Table 2), with higher minutes in active transport (75.4 ± 29.4 versus 36.6 ± 11.7, *P* = 0.03). In adults 19–44 years of age, there were no significant differences between MHO and MUO phenotypes for MPA (mins/day), MVPA (mins/day), muscle strength (times/week), usual daily activity, TV/video (hrs/day), computer (hrs/day), or the proportion of individuals meeting either physical activity or screen time recommendations.

For obese adults aged 45–85 years of age, MHO engaged in more MPA mins/day (MHO versus MUO: 24.3 ± 2.7 versus 19.4 ± 1.7, *P* = 0.03). There were no significant differences between MHO and MUO for VPA, MVPA, total PA, active transportation (% engaging in active transport and mins/week), usual daily activity, TV/video computer time, or proportion meeting recommendations for physical activity or screen time (Table 2).

In multivariable logistic regression analyses, after adjustment for relevant demographic characteristics, none of the physical activity or screen time variables were significantly associated with the MHO phenotype in adolescents (Table 3). In contrast to the findings among adolescents, adults 19–44 years of age, those with the MHO phenotype, had 85% higher odds of engaging in active commuting, had 17% higher odds of having more minutes in active transportation, and were 2.7 times more likely to engage in light activity versus sitting in usual daily activity. Furthermore, among adults 45–85 years of age, each mins/day of MPA was associated with a 36% higher odds of having the MHO phenotype. No other physical activity or screen time variables were significantly associated with the MHO phenotype in adults 19–44 years of age or 45–85 years of age (Table 3).

## 4. Discussion

Current findings from this obese U.S. sample suggest that certain aspects of physical activity, but not screen time, were associated with the MHO phenotype. These relationships varied by age, whereby the MHO phenotypes were associated with engaging in active transportation and usual daily activity in adults 19–44 years of age and higher levels of MPA in adults 45–85 years of age. There were no associations between MHO phenotype and physical activity among adolescents, and no associations between screen time and cardiometabolic phenotypes in either adolescents or adults. 

Previous studies of adult physical activity in obese individuals have not found differences between MHO and MUO adults [1, 13, 37, 38]; however, these studies were performed in postmenopausal, sedentary women [1, 13], South African Black women [37], or Koreans [38], limiting their generalizability to U.S. adults and adolescents. Other population studies in both men and women have shown that higher levels of total physical activity were associated with the MHO phenotype in the U.S. [9] and Switzerland [14]. However, the current study adds to this knowledge base by examining the associations between physical activity and sedentary behavior across different age groups (adolescents and adults 19–44, 45–85 years) and by differentiating between aspects of physical activity (types and intensities of physical activity, usual daily activity, active transportation). 

Although among adults aged 19–44 years of age, a greater percentage of individuals who engaged in active transportation had the MHO phenotype, and this did equate to a significantly greater amount mins/week of active transportation. It has been previously noted that active transportation can contribute to overall physical activity levels [39], and this may have also influenced our estimate of usual daily activity which indicated greater amounts of light usual daily activity in MHO when compared to MUO for adults 19–44 years. Levels of active transportation are also associated with cardiovascular risk factors and weight status [19, 21] and may represent a potential target relevant for intervention in this age group.

In the current study, screen time was not associated with MHO in either adolescents or adults. This is in contrast to previous work in adolescents [24, 26] and adults [23, 25, 40] that show that higher amounts of TV time are associated with elevated cardiovascular risk factors. It is important to acknowledge that we were limited in our sample size for these categorical screen time variables which may reduce our statistical power and precision to detect differences between obesity phenotypes. Thus, future studies need larger sample sizes to uncover possible differences for screen time variables. Although we did not observe an association between physical activity, screen time, and metabolic phenotypes among obese adolescents in this study, adolescence may be a critical time to intervene to prevent the emergence of adverse lifestyle behaviors in MUO that occur during adulthood. We know that physical activity [41–44], sedentary behaviors [41, 45], and specifically TV viewing [46] track from childhood/adolescence to adulthood. In addition, adverse cardiometabolic phenotypes emerge during adolescence, cardiometabolic risk factor levels track from childhood to adulthood [47], and the adult MHO phenotype can be predicted from childhood cardiometabolic risk factors [48]. 

NHANES data is representative of the U.S. population, and a strength of the current study is the generalizability of our results to the 17% of adolescents [49] and 33.8% of adults [50] who are obese. NHANES employs a cross-sectional design, and we cannot infer causation between physical activity/screen time variables and cardiometabolic risk phenotypes. Furthermore, despite our stratification into age groups, this cross-sectional study cannot infer associations across the lifespan from adolescence to adulthood. Future studies are needed to explore the longitudinal development of these obesity phenotypes over time and their relationships to these specific lifestyle behaviors over time. 

Self-report questionnaires were used for the assessment of both physical activity and screen time in this analysis. Adolescents and adults may be biased in reporting physical activity behaviors [51], which also may be relevant for screen time estimation. Although accelerometry, a more objective measure of activity was assessed in NHANES starting in 2003-2004, the sample size available was not adequate for the current analysis. Validity of physical activity questionnaires in comparison to objective measures may be limited, especially in adolescents [52], which may explain our lack of findings. Also, as previously mentioned, we were limited in our sample sizes which can limit statistical power to detect differences between MHO and MUO. Further, we were only able to capture a specific definition of sedentary behavior that involves sitting, TV/video viewing, and computer use. More global and specific measures of sedentary time involving any sitting or lying using more sophisticated methodology are needed to confirm our results. Thus, future studies should employ more objective measures of both physical activity and sedentary behavior to confirm results found in the current analysis. 

One of the challenges in the current literature for MHO is that standardized definitions for obesity phenotypes do not presently exist in either adolescents or adults. Previous studies have employed measures of insulin resistance/sensitivity [1, 7, 53–57], cardiometabolic clustering [9], adiposity [58–60], or a combination [6, 9, 61]. Velho et al. found that prevalence can vary depending on the definition used [14]. However, when Meigs et al. compared two models of obesity phenotypes, the insulin resistance versus cardiometabolic clustering, similar findings were found when relating to development of cardiovascular disease and type II diabetes in adults from the Framingham Offspring Study [8]. Durward et al., also compared various definitions for cardiometabolic risk and found that all definitions showed an increased risk for mortality [62]. These findings suggest that the definition chosen to define obesity phenotypes may not change the relationship to health-related outcomes. To our knowledge, we do not know similar studies that examine these relationships in adolescents. We chose to use a definition that was previously utilized to establish prevalence of obesity phenotypes in adolescents from NHANES [5]. A cardiometabolic clustering definition may be more clinically relevant since cardiovascular risk factors are routinely measured in primary care. Further, the use of an absolute cutpoint for the individual cardiometabolic risk factors allows a quick and easy determination of the obesity phenotypes. However, future studies are needed to explore the optimal definitions for adolescents and adults, and future recommendations for a standardized definition should be made.

 Despite these limitations, we are able to examine the association of physical activity and screen time with metabolic phenotypes in a U.S. sample of obese adolescents and adults. This study expands the extant literature by comparing varying intensities of physical activity, including MPA and VPA intensities, as well as other forms and domains of physical activity such as active transportation, strength training, and usual daily activity. In addition, we were able to report screen time for both adolescent and adults, increasing our understanding of potential intervention targets concerning those with the MUO phenotype. 

In conclusion, the current study provides some evidence that physical activity, but not sedentary behavior, is associated with cardiometabolic phenotypes among adults. In contrast, physical activity and sedentary behavior do not appear to be associated with MHO during adolescence. Thus, physical activity may represent a useful intervention target in obese adults to reduce cardiometabolic risk. 

## Figures and Tables

**Table 1:** Demographic and cardiometabolic characteristics* of metabolically healthy obese (MHO)^+^ and metabolically unhealthy obese (MUO) phenotypes in adolescents and adults.

	Adolescents 12–18 years (*n* = 225)			Adults 19–44 years (*n* = 270)			Adults 45–85 years (*n* = 271)		
	MHO	MUO	*P* value	MHO	MUO	*P* value	MHO	MUO	*P* value
*n* (% of age group)	163 (68)	62 (32)		152 (54)	118 (45)		64 (24)	207 (76)	

Demographic and cardiometabolic variables									
Age (yrs)	14.6 ± 0.2	15.1 ± 0.5	0.32	30.8 ± 0.6	34.8 ± 0.8	**<0.0001**	55.2 ± 1.1	56.6 ± 0.8	0.26
Male *n* (%)	67 (42)	40 (69)	**0.006**	63 (43)	66 (59)	0.05	38 (64)	100 (46)	0.11
Ethnicity *n* (%)									
Non-Hispanic white	35 (58)	19 (67)	0.15	50 (57)	48 (67)	0.22	36 (79)	126 (84)	0.61
Non-Hispanic black	66 (20)	11 (7)		55 (21)	33 (14)		15 (11)	41 (8)	
Hispanic	50 (12)	29 (16)		33 (10)	24 (8)		9 (4)	34 (4)	
Others	12 (10)	3 (9)		14 (12)	13 (12)		4 (7)	6 (4)	
Menopause *n* (% women)	N/A	N/A		4 (7)	6 (10)	0.54	26 (60)	84 (78)	0.08
BMI (kg/m^2^)	32.2 ± 0.4	32.5 ± 0.9	0.79	34.6 ± 0.4	36.6 ± 0.5	**0.004**	34.5 ± 0.5	35.0 ± 0.5	0.44
BMI *z*-score	2.1 ± 0.03	2.1 ± 0.05	0.33	N/A			N/A		
Triglycerides (mg/dL)^*∧*^	92.7 ± 4.1	168.1 ± 13.4	**<0.0001**	107.5 ± 4.3	201.5 ± 11.7	**<0.0001**	105.1 ± 8.2	187.1 ± 10.4	**<0.0001**
HDL C (mg/dL)	49.6 ± 1.1	36.5 ± 0.8	**<0.0001**	51.5 ± 1.0	42.3 ± 1.1	**<0.0001**	59.2 ± 1.6	49.0 ± 1.1	**<0.0001**
Systolic BP (mmHg)	111.9 ± 1.0	119.0 ± 2.1	**0.007**	116.6 ± 0.9	123.4 ± 1.3	**0.0003**	121.2 ± 1.2	130.0 ± 1.2	**<0.0001**
Diastolic BP (mmHg)	60.4 ± 1.0	61.9 ± 2.0	0.5	69.9 ± 1.1	77.0 ± 1.0	**<0.0001**	72.8 ± 1.4	73.4 ± 0.9	0.51
Fasting glucose (mg/dL)	92.1 ± 0.7	100.3 ± 2.4	**0.002**	93.4 ± 0.6	102.7 ± 0.9	**<0.0001**	93.5 ± 1.2	107.5 ± 1.2	**<0.0001**

*Mean ± SE or *n* (%).

^+^Obesity among adolescents defined by age/sex-specific BMI >95% percentile [31]; obesity among adults defined by BMI ≥30.0 kg/m^2^; MHO: 0, 1 abnormal cardiometabolic risk factor; MUO: ≥2 abnormal cardiometabolic risk factors.

^∧^Log transformed for *t*-test analysis.

NA: not applicable.

**Table 2:** Unadjusted* physical activity and screen time between MHO and MUO phenotypes among adolescents and adults.

	Adolescents 12–18 years			Adults 19–44 years			Adults 45–85 years		
	MHO	MUO	*P* value	MHO	MUO	*P*-value	MHO	MUO	*P* value
Physical activity									
Moderate PA^*∧*^ (min/day)	25.2 ± 3.7	24.9 ± 4.1	0.77	23.7 ± 3.0	22.1 ± 2.0	0.99	24.3 ± 2.7	19.4 ± 1.7	**0.03**
Vigorous PA^*∧*^ (min/day)	30.9 ± 6.7	26.0 ± 4.1	0.55	15.1 ± 1.9	8.7 ± 1.6	**0.01**	5.8 ± 1.7	7.1 ± 1.3	0.64
MVPA^*∧*^ (min/day)	56.1 ± 9.4	51.0 ± 6.4	0.55	38.8 ± 3.9	30.8 ± 2.3	0.16	30.1 ± 3.3	26.5 ± 2.3	0.12
PA^*∧*^ (MET ∗ min/day)	359.6 ± 66.1	319.8 ± 43.3	0.5	205.0 ± 20.9	150.2 ± 14.0	0.07	130.5 ± 16.6	126.0 ± 12.7	0.16
Meeting PA recommendations^"^ *n* (%)	52 (29)	20 (36)	0.46	67 (44)	45 (41)	0.68	22 (35)	76 (33)	0.87
Muscle strength (times/week)	2.4 ± 0.6	1.5 ± 0.4	0.54	1.2 ± 0.2	1.2 ± 0.2	0.59	1.0 ± 0.3	0.7 ± 0.1	0.53
Active transport *n* (%)	98 (48)	30 (44)	0.65	55 (37)	32 (23)	**0.01**	12 (20)	50 (20)	0.57
Active transport^*∧*^ mins/wk	187.1 ± 81.3	217.1 ± 90.0	0.73	75.4 ± 29.4	36.6 ± 11.7	**0.03**	14.3 ± 6.2	33.5 ± 7.5	0.33
Usual daily activity *n* (%)									
Sit	NA			27 (19)	23 (23)	0.16	14 (23)	46 (22)	0.99
Walk				77 (49)	62 (51)		39 (59)	126 (58)	
Light activity				36 (25)	19 (14)		9 (14)	27 (16)	
Heavy activity				12 (7)	14 (12)		2 (4)	8 (4)	

Screen time									
TV/video (# hours/day)									
≤1 hr/day	47 (33)	25 (37)	0.25	42 (34)	45 (40)	0.71	20 (30)	49 (27)	0.59
2-3 hr/day	80 (44)	17 (29)		68 (44)	46 (37)		37 (59)	106 (55)	
4+ hr/day	36 (23)	20 (34)		42 (22)	27 (23)		7 (10)	52 (18)	
Computer (# hours/day)									
≤1 hr/day	126 (75)	42 (55)	0.14	119 (82)	87 (71)	0.15	53 (84)	179 (84)	0.51
2-3 hr/day	30 (19)	11 (30)		22 (13)	22 (22)		8 (13)	17 (10)	
4+ hr/day	7 (7)	9 (15)		11 (67)	9 (7)		3 (3)	11 (7)	
Meeting screen time recommendations** n (%)	44 (30)	21 (27)	0.75	45 (38)	45 (36)	0.87	23 (38)	64 (30)	0.37

*Mean ± SE or *n* (%).

^"^≥60 mins /day MVPA for adolescents; ≥30 mins/day adults.

**≤2 hrs screen time per day as recommended for children and adolescents by American Academy of Pediatrics.

^∧^Variable log transformed for analysis.

N/A: not available.

**Table 3:** Associations of MHO phenotypes* with physical activity and screen time, among adolescents and adults^*∧*^.

	Adolescents (12–18 yrs)	Adults (19–44 yrs)	Adults (45–85 yrs)
	OR (95% CI)	OR (95% CI)	OR (95% CI)
Physical activity			
Moderate PA^+^ (min/day)	0.99 (0.74–1.32)	1.08 (0.90–1.30)	**1.36 (1.04**–**1.78)**
Vigorous PA^+^ (min/day)	1.0 (0.77–1.30)	1.21 (0.96–1.52)	0.97 (0.75–1.26)
MVPA^+^ (min/day)	0.96 (0.64–1.44)	1.21 (0.91–1.60)	1.29 (0.95–1.77)
PA^+^ (MET ∗ min/day)	0.95 (0.66–1.37)	1.22 (0.94–1.59)	1.25 (0.93–1.69)
Meeting PA recommendations^"^	0.85 (0.30–2.39)	1.37 (0.72–2.64)	1.10 (0.54–2.25)
Muscle strength (#/wk)	1.19 (0.55–2.56)	0.82 (0.51–1.32)	1.36 (0.64–2.92)
Active transport (%)	1.21 (0.57–2.54)	**1.85 (1.11**–**3.10)**	0.74 (0.38–1.45)
Active transport^+^ (mins/wk)	1.04 (0.91–1.19)	**1.17 (1.02**–**1.35)**	0.92 (0.78–1.09)
Usual daily activity			
Sit	N/A	1 (Reference)	1 (Reference)
Walk		1.05 (0.43–2.55)	0.92 (0.41–2.06)
Light activity		**2.70 (1.17**–**6.19)**	0.82 (0.22–2.96)
Heavy activity		0.53 (0.18–1.51)	0.89 (0.15–5.41)

Sedentary behavior			
TV/video (# hours/day)			
≤1 hr/day	1.45 (0.43–4.97)	1.42 (0.52–3.88)	1.48 (0.39–5.58)
2-3 hr/day	2.75 (0.99–7.63)	1.82 (0.85–3.88)	2.04 (0.65–6.43)
4+ hr/day	1 (Reference)	1 (Reference)	1 (Reference)
Computer (# hours/day)			
≤1 hr/day	1.86 (0.40–8.65)	1.91 (0.70–5.20)	2.40 (0.70–8.19)
2-3 hr/day	1.00 (0.14–6.93)	0.79 (0.26–2.39)	4.21 (0.74–23.74)
4+ hr/day	1 (Reference)	1 (Reference)	1 (Reference)
Meeting screen time recommendations**	1.12 (0.42–2.93)	1.43 (0.64–3.20)	1.09 (0.47–2.52)

*Reference: MUO; outcome: MHO.

^∧^Adjusted for age, gender, BMI (*z*-score in adolescents; kg/m^2^ in adults), race/ethnicity (non-Hispanic white, non-Hispanic black, Mexican American, and others: mixed and Hispanic), menopausal status, and NHANES wave (2003-2004, 2005-2006).

**≤2 hrs screen time per day as recommended for children and adolescents by American Academy of Pediatrics.

^+^Variable log transformed for analysis.

^"^≥60 mins /day MVPA for adolescents; ≥30 mins/day adults.

N/A: not available.
